# Single cell RNA-sequencing data generated from human pluripotent stem cell-derived lens epithelial cells

**DOI:** 10.1016/j.dib.2020.106657

**Published:** 2021-01-08

**Authors:** Rachel Shparberg, Chitra Umala Dewi, Vikkitharan Gnanasambandapillai, Liwan Liyanage, Michael D. O'Connor

**Affiliations:** a School of Medicine, Western Sydney University, Campbelltown, NSW 2560, Australia; b Garvan-Weizmann Centre for Cellular Genomics, Garvan Institute, UNSW Cellular Genomics Futures Institute, University of New South Wales, Sydney 2010, Australia; c School of Computer, Data and Mathematical Sciences, Western Sydney University, Campbelltown, NSW 2560, Australia

**Keywords:** Human lens epithelial cell, ROR1, Pluripotent stem cell, Micro-lens, Cataract, Single cell RNA-sequencing, Bioinformatics

## Abstract

Detailed transcriptomic analyses of differentiated cell populations derived from human pluripotent stem cells is routinely used to assess the identity and utility of the differentiated cells. Here we provide single cell RNA-sequencing data obtained from ROR1-expressing lens epithelial cells (ROR1e LECs), obtained via directed differentiation of CA1 human embryonic stem cells. Analysis of the data using principal component analysis, heat maps and gene ontology assessments revealed phenotypes associated with lens epithelial cells. These data provide a resource for future characterisation of both normal and cataractous human lens biology. Corresponding morphological and functional data obtained from ROR1e LECs are reported in the associated research article “A simplified method for producing human lens epithelial cells and light-focusing micro-lenses from pluripotent stem cells “ (Dewi et al., 2020).

## Specifications Table

**Table:** 

Subject	Biology
Specific subject area	Molecular Biology, Cell Biology, Human Pluripotent Stem Cells, Lens Epithelial Cells, Single Cell Transcriptomics
Type of data	Table Figure
How data were acquired	Stem cell culture and differentiation. RNA-Seq (10X Genomics single cell 3′ mRNA-prep kit and Illumina NextSeq 500). Transcriptomic data analyses (Seurat guided clustering suite version 3; Bioconductor R package; DAVID gene ontology).
Data format	Raw fastq files
Parameters for data collection	Human pluripotent stem cells were differentiated to lens epithelial cells using established methods (Murphy et. al, 2018). ROR1e LECs were harvested on day 16 and expanded for a further 7 days in optimised medium (Murphy et. al, 2018). Cells were collected 7 days after harvest and immediately processed for scRNA-seq.
Description of data collection	Total RNA was isolated from ROR1e LECs. cDNA library was constructed and scRNA-seq performed. Bioinformatics performed using Seurat guided clustering suite version 3.
Data source location	School of Medicine, Western Sydney University, Campbelltown, Australia
Data accessibility	ArrayExpress accession number E-MTAB-9178 https://www.ebi.ac.uk/arrayexpress/experiments/E-MTAB-9178/
Related research article	Umala Dewi et al, 2020, A simplified method for production of human lens epithelial cells and light-focusing micro-lenses from pluripotent stem cells, Exp. Eye Res. Experimental Eye Research. doi.org/10.1016/j.exer.2020.108317.

## Value of the Data

• These single cell RNA-sequencing profiles, obtained from lens epithelial cells derived from human embryonic stem cells, provide insights into the transcriptional heterogeneity of human lens cell cultures.
• Exploration of these data will provide molecular insights into regulation of gene expression in human lens epithelial cells that will be of use to researchers investigating lens and cataract development.
• These data can be further analysed to better understand lens transcriptional regulators, and to provide molecular hypotheses to guide functional investigations of normal lens biology and cataract initiation.

## 1 Data Description

**Fig. 1:**
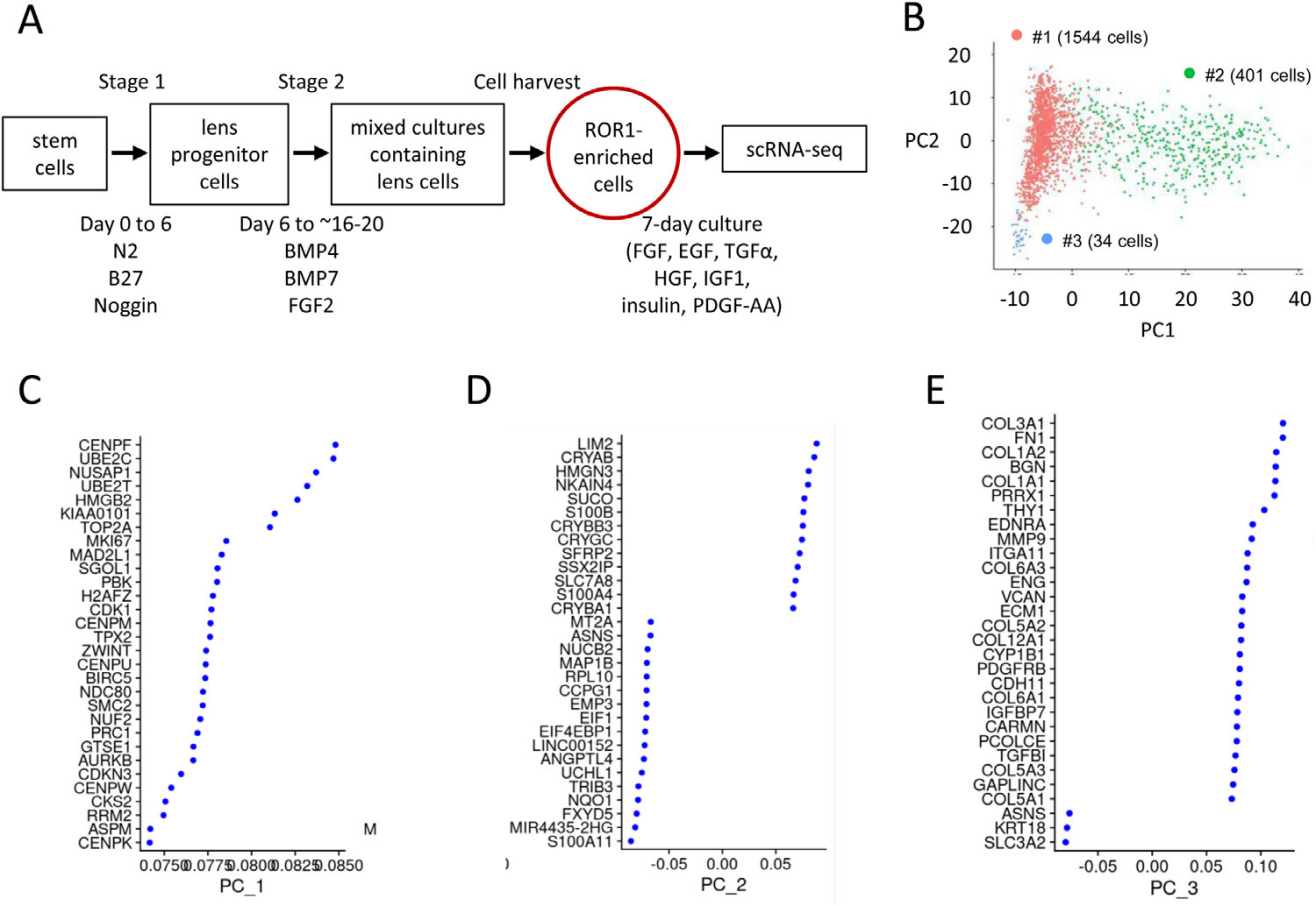
Single cell RNA sequencing analysis of ROR1e LECs. (A) Schematic diagram outlining generation of ROR1e LECs from CA1 human embryonic stem cells. (B) PCA analysis revealed three cell clusters within the ROR1e LEC scRNA-seq data. (C-E) Genes that show high cell-cell variability across the data set in the top three principal components.

Here we report initial scRNA-seq data analyses for human, pluripotent stem cell-derived, ROR1e LECs. These LECs were generated from CA1 human embryonic stem cells as outlined in Fig. 1. The culture protocol included 16 days guided differentiation of the stem cells to lens cells, followed by harvesting of ROR1e LECs and then an additional 7-days culture in an optimised LEC medium [1]. Phenotypic characterisation of similar ROR1e LEC populations demonstrated: they are morphologically-similar to antibody-purified ROR1^+^ LECs; they express expected LEC crystallin proteins; and they can be aggregated to generate light-focusing micro-lenses that have similar morphology and protein expression to primary human lenses [2].

### 1.1 Principal component analyses

The ROR1e LECs were dissociated immediately prior to processing for scRNA-seq. RNA was extracted from the cultured LECs and processed using a 10X Genomics single cell 3′ mRNA-prep kit. Sequencing was performed using an Illumina NextSeq 500 sequencer. The scRNA-seq data can be accessed via Array Express accession E-MTAB-9178. Analysis using the Seurat guided clustering suite yielded 1979 cells, with a total of 17,944 genes expressed. Assessment via principal component analysis (PCA) revealed three distinct cell clusters (Fig. 1B). Together, clusters 1 and 2 accounted for 98.3% of the total cell population (78% and 20.3%, respectively). Cluster 3 accounted for 1.7% of the total cell population. The main genes responsible for the observed variation between the cells are shown (Fig. 1C–E).

### 1.2 Heat map analysis of critical lens genes

**Fig. 2:**
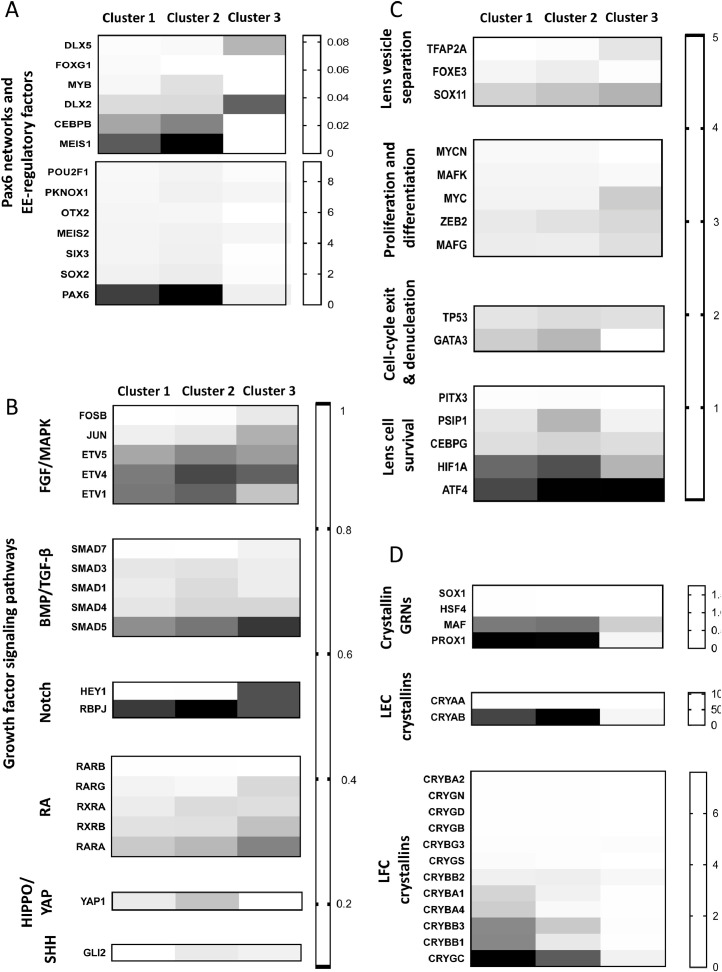
Expression of key lens genes. Heat maps showing average expression per cell in each cluster for important lens-related genes, with gene groupings based on gene function.

Heat map analyses were used to investigate expression of critical lens genes in each of the three cell clusters. The initial analysis examined expression of 65 genes known to be required for lens development. This included growth factor signaling genes, crystallin genes and their regulators, proliferation and cell survival genes, and various transcription factors [3] (Fig. 2). Clusters 1 and 2 expressed similar levels of all 65 genes – including high expression of critical lens genes such as PAX6 and CRYAB – while some genes had reduced expression in cluster 3. Overall, these data suggest the ROR1e cells have a lens phenotype.

### 1.3 Analysis of the top 20 cluster marker genes

**Fig. 3:**
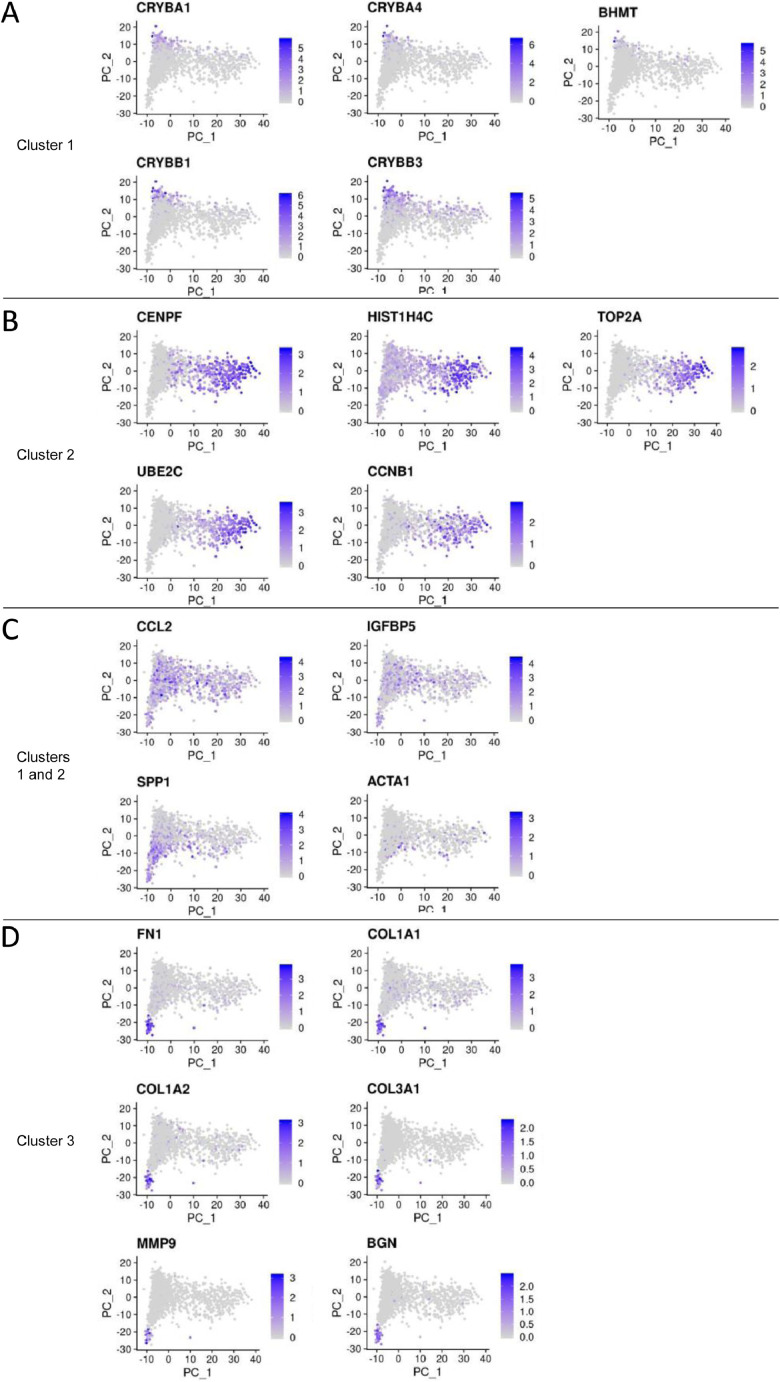
Distribution of the top 20 most-differentially expressed genes. (A-D) The top 20 differentially expressed genes overlayed on the PCA plot: 5 of the genes were associated with cluster 1 (A); 5 genes were associated with cluster 2 (B); 4 genes were associated with both clusters 1 and 2 (C); and 6 genes were associated with cluster 3 (D).

Assessment of the top 20 genes most differentially expressed across the 3 clusters (Fig. 3) showed 70% of these genes (14/20) were more highly expressed in Clusters 1 and/or 2, and 30% (6/20) more highly in Cluster 3. In particular: Cluster 1 had higher expression of crystallin genes associated with lens fibre cells (Fig. 3A); Cluster 2 had higher expression of some genes associated with cell proliferation such as CENPF, TOP2A, UBE2C and CCNB1 (Fig. 3B); and Cluster 3 had higher expression of some genes associated with epithelial-to-mesenchymal transition (Fig. 3D).

### 1.4 Analysis of cluster 1 marker genes

**Fig. 4:**
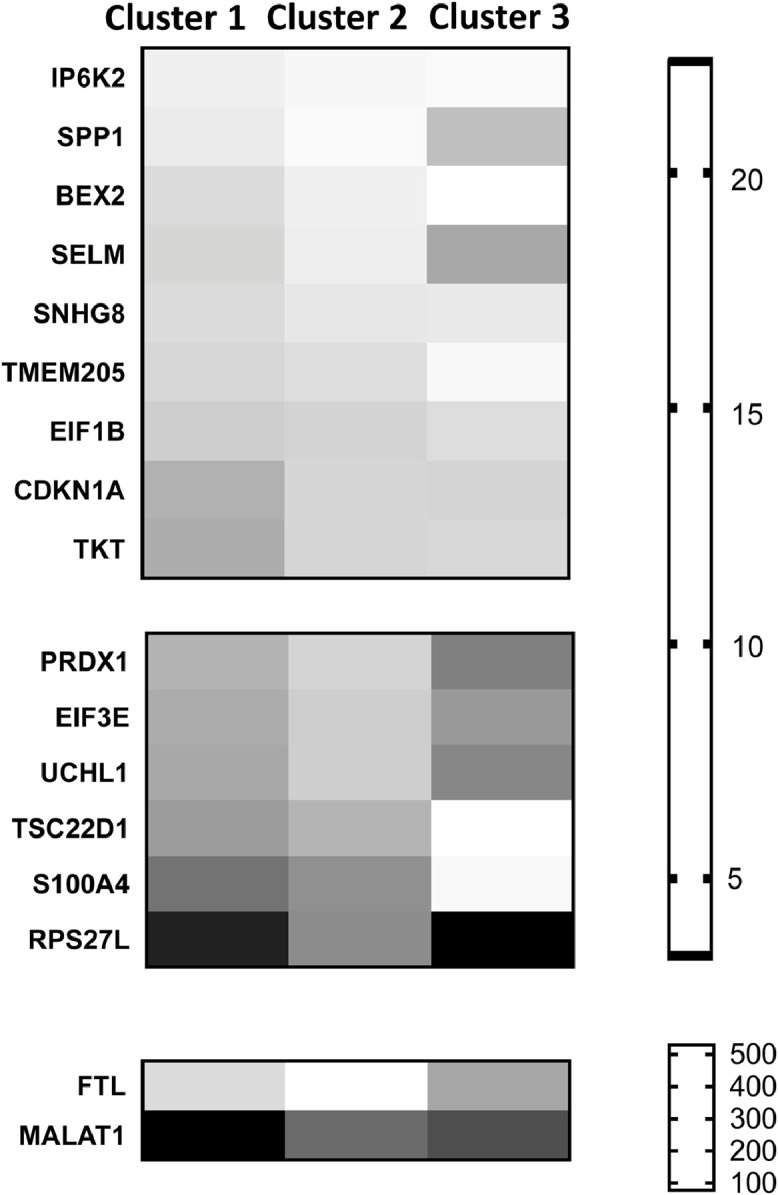
Heat maps of cluster 1 marker genes. The 17 marker genes that were significantly and positively associated with cluster 1 were grouped into three heatmaps based on average gene expression level per cell, then sorted based on lowest to highest expression in cluster 1.

Heat maps and GO analyses were then used to assess the 17 genes identified as marker genes for cluster 1. The heat map analysis showed cluster 1 marker genes (Fig. 4) were more similarly expressed in cluster 2 than in cluster 3 – suggesting cluster 1 is similar to cluster 2. No significant GO terms were associated with the cluster 1 marker genes. Comparison with published lens transcriptomic datasets revealed all the cluster 1 marker genes are expressed in adult human lenses [4] and/or mouse lenses [5] – including protein-coding and non-coding genes required for normal lenses (e.g., BEX2, TKT, SNHG8, MALAT1) [6].

### 1.5 Analysis of cluster 2 marker genes

**Fig. 5:**
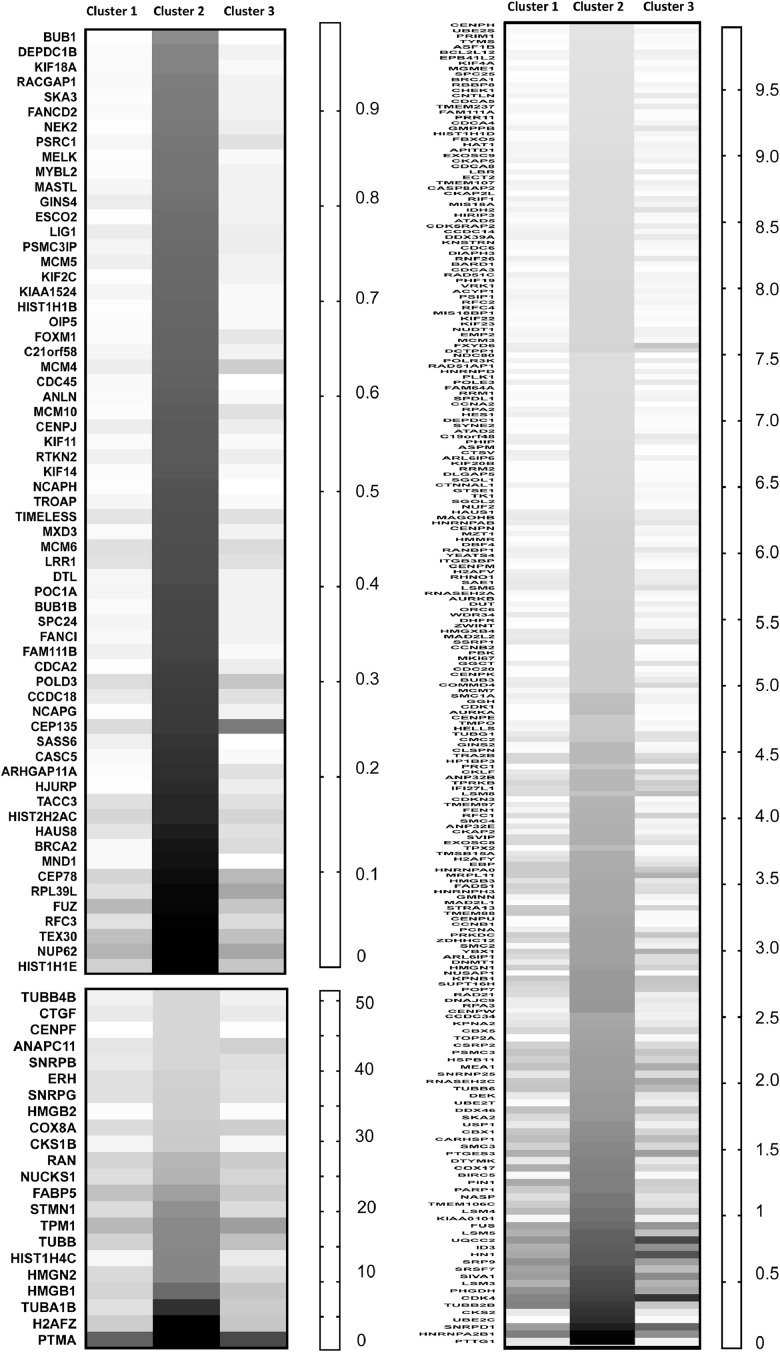
Heat maps of cluster 2 marker genes. The 314 marker genes that were significantly and positively associated with cluster 2 were grouped into three heatmaps based on average gene expression level per cell, then sorted based on lowest to highest expression in cluster 2.

For the 314 genes identified as markers of cluster 2 (Fig. 5), most were expressed much higher in cluster 2 compared to both clusters 1 and 3 – particularly for lowly-expressed cluster 2 marker genes. GO analysis of the cluster 2 marker genes identified terms relating to cell cycle and mitosis (Table 1). These data indicate cluster 2 contains cells that had higher expression of some proliferation-related genes when the cell population was captured.

**Table 1:** Top 20 significant GO terms associated with Cluster 2 markers.

### 1.6 Analysis of cluster 3 marker genes

**Fig. 6:**
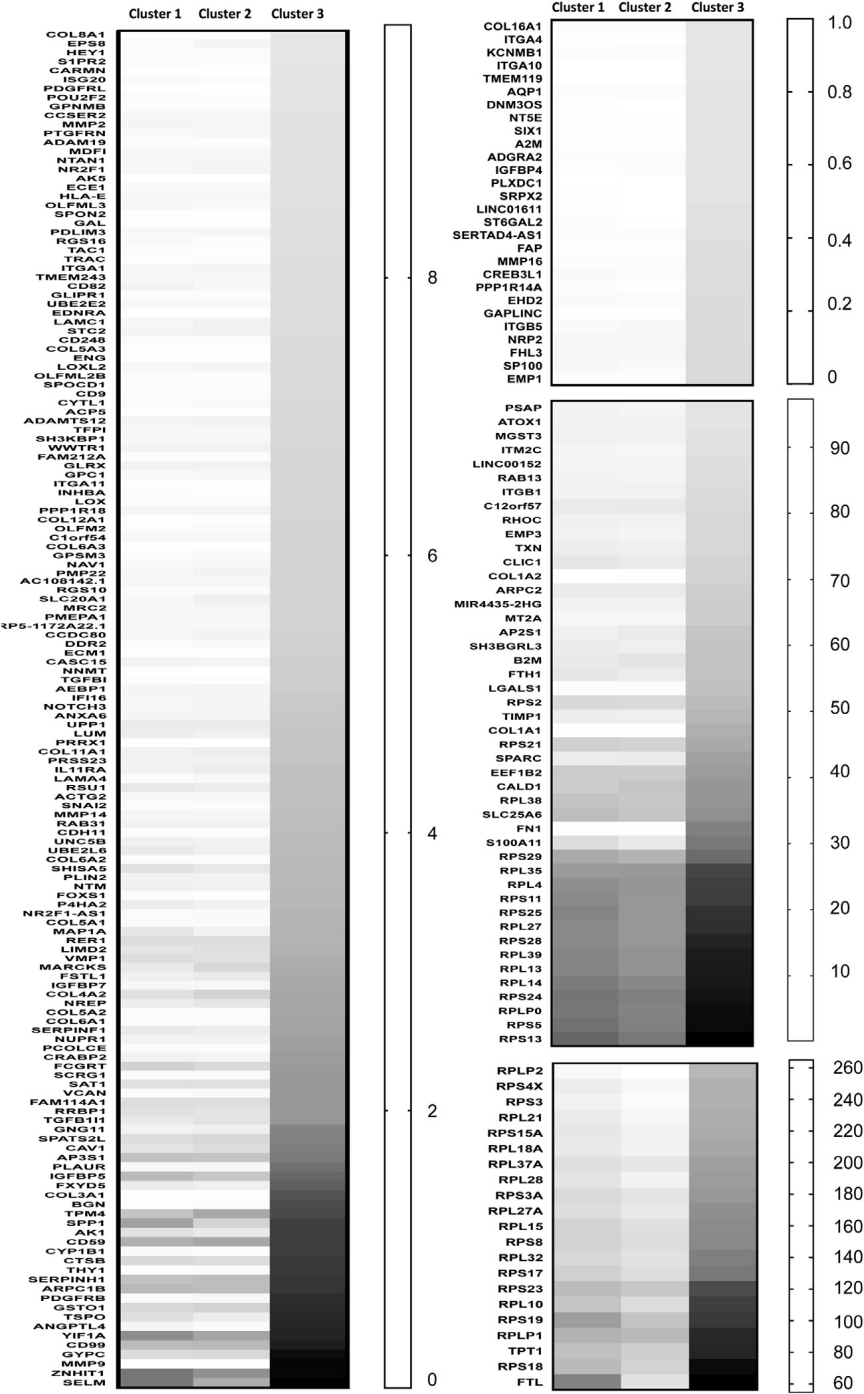
Heat maps of cluster 3 marker genes. The 245 marker genes that were significantly and positively associated with cluster 3 were grouped into four heatmaps based on average gene expression level per cell, then sorted based on lowest to highest expression in cluster 3.

For the 245 cluster 3 marker genes (Fig. 6), most were detected in clusters 1 and 2 but at lower levels. GO analysis of the cluster 3 marker genes identified terms relating to collagen metabolism and extracellular matrix degradation (Table 2). As the cluster 3 marker gene list includes fibronectin, MMP9 and collagen 1/3 genes (Fig. 3D), it is possible cluster 3 represents cells primed for epithelial-to-mesenchymal transition (though this cluster represents only 1.7% of the total population of ROR1e LECs).

**Table 2:** Top 20 significant GO terms associated with Cluster 3 markers.

### 1.7 Comparison of GO terms obtained from all expressed genes in each cluster

To gain a more detailed insight into the relative difference in transcriptomes between the three ROR1e LEC clusters, additional GO analyses were performed using the expressed genes for each cluster. When the expressed genes from all three cluster were combined, the GO terms identified were consistent with known lens biology. This includes GO terms relating to establishment of a polarised epithelium (a key feature of primary LECs), and terms related to growth factor signalling pathways required by LECs (e.g., FGF and WNT) [7]. When the expressed genes from each cluster were analysed separately, 15 of the top 20 GO terms were shared across all the clusters [2]. The first 12 of these 15 shared GO terms were the most statistically-significant GO terms for each of the three clusters (excluding generic terms). Strikingly, these shared GO terms related to establishment of a polarised epithelium, morphogenesis of an epithelium, FGF signalling and WNT signalling. A GO term relating to epithelial to mesenchymal transition was identified in the very small cluster 3. Overall these GO analyses are consistent with stem cell-derived ROR1e LECs being a highly purified population of human LEC-like cells. These findings are also consistent with morphological, lens function, proteomic and electron microscopy data obtained using ROR1e LECs [2].

## 2 Experimental Design, Materials and Methods

### 2.1 Generation of ROR1e LECs

CA1 human embryonic stem cells were obtained from A. Nagy [8]. Approval for their use was obtained from the Western Sydney University Human Research Ethics Committee (Australia). CA1 cells were maintained on Matrigel-coated plates (Corning, Sydney, Australia) in mTeSR1 (StemCell Technologies, Sydney, Australia) as previously described [9,10]. Generation of heterogeneous differentiation cultures containing lens cells occurred in: DMEM:F12 (Thermo Fisher Scientific, Sydney Australia) containing 500 ng/mL noggin and 10 nM SB431542 (6 days); followed by DMEM:F12 containing 20 ng/mL BMP4, 20 ng/mL BMP7 and 100 ng/mL FGF2 [2]. Growth factors were sourced from Miltenyi Biotec and Peprotech (Sydney, Australia). ROR1e LECs were harvested using TrypLE (Thermo Fisher Scientific), filtered through a 40-micron cell strainer (Interpath Services, Sydney, Australia), centrifuged (300 g, 5 min) and counted, then resuspended in medium containing 10 ng/mL FGF2, 5 ng/mL EGF and TGFα, 10 ng/mL HGF, 10 ng/mL IGF1, 10 μg/mL insulin and 10 ng/mL PDGF-AA [1]. After 7 days, ROR1e LECs were harvested using TrypLE and immediately processed for scRNA-seq.

### 2.2 Single cell RNA sequencing

The ROR1e LECs were processed using a 10X Genomics single cell 3′ mRNA-prep kit as per the manufacturer instructions (part numbers PN-120267, PN-1000009 and PN-120262). Sequencing was performed by the Ramaciotti Centre for Genomics (Sydney, Australia) using an Illumina NextSeq 500 (NextSeq Control Software v 2.2.0.4 / Real Time Analysis 2.4.11), with 150 cycles as follows: 26bp (Read 1), 98bp (Read 2) and 8bp (Index). Read alignment, filtering, barcode counting, and UMI counting were performed using CellRanger (v3.1.0) and FASTQ files. The reads were aligned to Genome Reference Consortium Human Build 38.

### 2.3 Bioinformatic analyses

Processed scRNA-seq data were analysed using R and the Seurat guided clustering suite version 3 [11]. The data was filtered to include cells with unique feature counts between 200 and 6000 and with <10% mitochondrial counts. Global-scaling normalization (LogNormalize) was used to normalizes the feature expression measurements for each cell. A subset of features exhibiting high cell-to-cell variation was calculated using the module FindVariableFeatures. A standard, pre-processing linear transformation scaling was applied (ScaleData function). The data then underwent linear dimension reduction via PCA using the previously determined variable genes. Marker genes were identified for each cell cluster using ROC analysis and taking the default value of 0.25 log(feature count) threshold-within FindAllMarkers. Identified clusters of genes were visualised using PCA plots where individual genes were overlayed on the plot. GO analyses were performed using the Functional Annotation tool of the DAVID Bioninformatics Resources, version 6.8 [12], [13], [14]. For each cluster, GO analysis was performed using both all expressed genes, and genes with average expression count of ≥1 per cell. The top 20 GO terms are reported for each cluster based on Benjamini p-values, excluding generic GO terms related to common cellular processes (e.g., cell cycle, macromolecule processing, respiration, protein processing, regulation of apoptosis, etc.).

## Ethics Statement

Approval for this study was provided by the Western Sydney University Human Research Ethics Committee (Australia).

## CRediT Author Statement

**Rachel Shparberg:** Visualisation, Writing – Original draft preparation, Writing – Reviewing and Editing **Chitra Umala Dewi:** Methodology, Investigation, **Vikkitharan Gnanasamandapillai:** Methodology, **Liwan Liyanage:** Data curation, Methodology, Software, Formal analysis, Validation **Michael O'Connor:** Conceptualization, Methodology, Resources, Supervision, Writing – Original draft preparation, Writing – Reviewing and Editing, Project Administration, Funding acquisition

## Declaration of Competing Interest

The authors declare that they have no known competing financial interests or personal relationships which have or could be perceived to have influenced the work reported in this article.
